# Improvement in Spine Pain, Functional Performance, and Quality of Life in a 26-Year-Old Male With a Failed Spine Fusion Surgery After Chiropractic BioPhysics® Structural Spinal Rehabilitation: A Case Report With a Six-Month Follow-Up

**DOI:** 10.7759/cureus.71544

**Published:** 2024-10-15

**Authors:** Jason W Haas, Curtis Fedorchuk, Douglas F Lightstone, Paul A Oakley, Deed E Harrison

**Affiliations:** 1 Research, Chiropractic BioPhysics (CBP) Non-profit, Inc., Windsor, USA; 2 Chiropractic Biophysics, Institute for Spinal Health and Performance, Cumming, USA; 3 Chiropractic, Institute for Spinal Health and Performance, Cumming, USA; 4 Kinesiology and Health Science, York University, Toronto, CAN; 5 Chiropractic, Innovative Spine and Wellness, Newmarket, CAN; 6 Research, Chiropractic BioPhysics (CBP) Non-profit, Inc., Eagle, USA

**Keywords:** cervical lordosis, chiropractic biophysics, instrumented spine fusion, neck pain, persistent spinal pain syndrome, radiography, spine surgery, upper cervical spine

## Abstract

Neck pain (NP) is a leading cause of disability and can be a consequence of failed cervical spine surgeries. Articles showing successful conservative therapies after a failed surgery in the cervical spine are very rare. A 26-year-old male reported six years of worsening and disabling NP. The short-form 36-question health status questionnaire revealed a decrease in quality-of-life scores, with a physical component score (PCS) of 25.2 and a mental component score (MCS) of 29.9, compared to the normal scores of 46.8 and 52.8, respectively. Grip strength measured 36.7 kg on the left and 37.1 kg on the right (normal range: 45-52 kg). Radiography revealed cervical hypolordosis (absolute rotation angle, ARA, C2-C7) and anterior head translation (T_z_ C2-C7) measuring -14.6° and 20.6 mm (ideal is -42° and 0 mm). Chiropractic BioPhysics^®^ (CBP^®^) (CBP Non-Profit, Inc., Eagle, ID) spinal rehabilitation sessions were administered involving Mirror Image^® ^(CBP Non-Profit, Inc.) spinal exercises, traction, and adjustments to correct cervical spinal alignment. Following 30 treatments over nine weeks, the patient reported near-resolution of initial symptoms, discontinued pain medications, and improved quality of life. Posttreatment outcomes included the following: improvement in PCS (45.6) and MCS (37.1), normalized grip strength on the left (45.3 kg) and right (49.4 kg), and improvement in ARA C2-C7 (30.1°) and T_z_ C2-C7 (15.6 mm). After six months without treatment, a follow-up examination showed sustained improvements in symptoms and outcome measures, including ARA C2-C7 (30.9°) and T_z_ C2-C7 (10.6 mm). Failed cervical spine surgeries and persistent spine pain syndrome can occur with devastating consequences. CBP^®^ may be an effective, conservative approach to help improve pain and disability in patients with poor surgical outcomes and abnormal spinal alignment.

## Introduction

Neck pain (NP) is a major cause of pain and disability and is a leading reason for the inability to work, significantly reducing overall quality of life [1,2]. Failed neck surgery results from upper cervical fusion surgeries [3]. Surgical complications include infection, loosened hardware, poor bone purchase, wrong patient selection, poor alignment, mental health (MH) issues, poor cervical spine mobility, and more [4-6]. Complications, poor patient-reported outcomes, abnormal findings on objective measures such as imaging, laboratory analysis, and balance often lead the patient to seek continued care after the surgery [7,8].

This is the case of a patient suffering from worsened chronic neck pain (CNP) following unsuccessful previous interventions, including medication, physical therapy, and upper cervical posterior approach spine fusion at the level of C1-C2. Over a short period of time, the pain worsened, expanded, and led to spine pain and radicular symptoms consistent with persistent spine pain syndrome (PSPS) and worsening PROs. This is consistent with previous reports of PSPS and led the patient to seek alternative treatment for his pain and disability, spine alignment, and postural problems. The patient ended up experiencing improvement in pain, health-related quality of life (HRQoL), functional performance, and spinal alignment and sagittal balance following Chiropractic BioPhysics® (CBP®) (CBP Non-Profit, Inc., Eagle, ID) structural spinal rehabilitation treatment protocols. This is particularly important in the current treatment landscape for NP and associated conditions as it can potentially provide treatment options for failed surgical and conservative interventions.

## Case presentation

Patient presentation and history

In September 2020, a 26-year-old male (height: 188.0 cm, weight: 99.8 kg) presented in a rehabilitation facility in Cumming, GA, seeking treatment for CNP. This facility specializes in the correction of spine alignment, causing pain and suffering. A long history of suffering was reported, with the patient reporting several prior interventions beginning with traditional chiropractic and physical therapy and eventually leading to surgical consultation and the performance of upper cervical fusion surgery. Given the failure of these prior interventions to ameliorate the pain and suffering, the patient sought treatment from a provider specializing in the correction of abnormal spine alignment. The patient reported constant sharp, stabbing, disabling NP that was rated 9-10/10 on the NP numeric rating scale (NRS) scale of 0-10, where 0 is no pain and 10 is maximum pain. The patient reported that the pain began eight years ago and gradually worsened, leading them to seek surgical intervention to relieve the pain and suffering. The patient reported that this pain was not alleviated following the upper cervical spine fusion. The subject endured two more years with pain, disability, and loss of function before reporting to the current facility. He reported his NP and range of motion (ROM) were worsening. He stated that prescription and over-the-counter (OTC) medication and ice therapy were not alleviating his problems. He reported that he was seeking care as his condition worsened following the upper cervical fusion surgery, and the subject was not interested in further surgery.

The subject reported frequent low energy and weakness, inability to exercise, self-medication with alcohol, and was taking Synthroid, clonazepam, tramadol, and baclofen at the time of the initial exam. The patient felt the medications were becoming less effective in managing pain, and he reported frequently having severe fatigue following taking the medications. The pain was causing depression, and the symptoms were spreading throughout the spine into the middle and lower back, which he rated 7-8/10 on the NRS.

Physical examination: objective and radiographic findings

The physical examination revealed NP, mid-back pain, and low back pain with cervical compression orthopedic testing. Grip strength testing revealed 37.1 kg on the right and 36.7 kg on the left (normal for sex and age is 45-52 kg) [9]. Visual ROM analysis showed restrictions with severe pain in all ROM with notable significant loss of cervical extension. Short-form 36-question health status questionnaire (SF-36) demonstrated reduction in physical function (PF) at 30/100, role limitations due to physical health (RP) at 0/100, role limitations due to emotional problems (RE) at 33.3/100, vitality energy/fatigue (VT) at 15/100, MH at 32/100, social functioning (SF) at 12.5/100, bodily pain (BP) at 10/100, general health (GH) at 30/100, and change in health status (ΔH) at 0/100 (Table 1).

**Table 1:** Pretreatment, posttreatment, and six-month follow-up SF-36 Health-Related Quality-of-Life Scale scores Measurement on the SF-36, where 0 is considered no function and 100 is a perfect function PF: physical functioning; RP: role limitations due to physical health; RE: role limitations due to emotional problems; VT: vitality (energy/fatigue); MH: mental health; SF: social functioning; BP: bodily pain; GH: general health; ΔH: change in health; PCS: physical component score; MCS: mental component score; SF-36: short-form 36-question health status questionnaire

SF-36 quality-of-life scales	Normative mean scale scores	Pre-CBP^®^ treatment exam in September 2020	Post-CBP^®^ treatment exam in November 2020	Six-month Post-CBP^®^ follow-up exam in May 2021
PF	72.0	30.0	70.0	65.0
RP	81.0	0.0	50.0	50.0
RE	81.0	33.3	50.0	50.0
VT	61.0	15.0	60.0	55.0
MH	81.0	32.0	52.0	64.0
SF	83.0	12.5	37.5	37.5
BP	75.0	10.0	67.5	67.5
GH	72.0	30.0	55.0	50.0
ΔH	84.0	0.0	75.0	75.0
PCS	46.8	25.1	45.6	42.5
MCS	52.8	29.9	37.1	40.3

**Figure 1:**
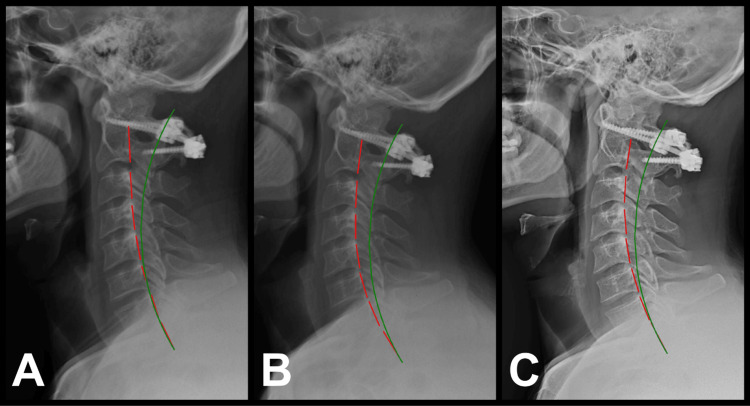
Pretreatment, posttreatment, and six-month post-follow-up neutral lateral cervical radiographs measuring cervical curvature. The red lines represent the actual posterior tangent lines of the C2-T1 vertebrae using the Harrison posterior tangent method on the lateral cervical radiograph, and the green lines represent the ideal spine model. (A) Pre-CBP® treatment neutral lateral cervical radiograph on September 11, 2020, with a cervical curvature ARA C2-C7 measuring -14.6° (ideal is -42°, normal is -34°, and pain threshold is -20°) and anterior head translation Tz C2-C7 measuring 20.6 mm (ideal is 0 mm, normal is 10 mm, and pain threshold is 20 mm). (B) Post-CBP® treatment neutral lateral cervical radiograph on November 12, 2020, with ARA C2-C7 measuring -30.1° and Tz C2-C7 measuring 15.6 mm. (C) Six-month follow-up neutral lateral cervical radiograph on May 12, 2021, with ARA C2-C7 measuring -30.9° and Tz C2-C7 measuring 10.6 mm CBP®: Chiropractic BioPhysics®; ARA: absolute rotation angle; Tz: anterior head translation

Standing radiographs were taken in accordance with Medicare laws in the United States. The images were taken with the patient in an upright, weight-bearing, and neutral position. Radiography assessment demonstrated significant abnormalities of sagittal cervical curvature from absolute rotation angle (ARA C2-C7) using the Harrison posterior tangent method and anterior head translation (T_z_ C2-C7) with PostureRay® radiographic digitization software (PostureCo, Inc., Trinity, FL). ARA C2-C7 was reduced by 55%, measuring 14.6° (ideal is -42°, normal is -34°, and pain threshold is -20°) [10]. T_z_ C2-C7 was measured at 20.6 mm (ideal is 0 mm, normal is 10 mm, and pain threshold is 20 mm) (Figure 1A) [10].

**Figure 2:**
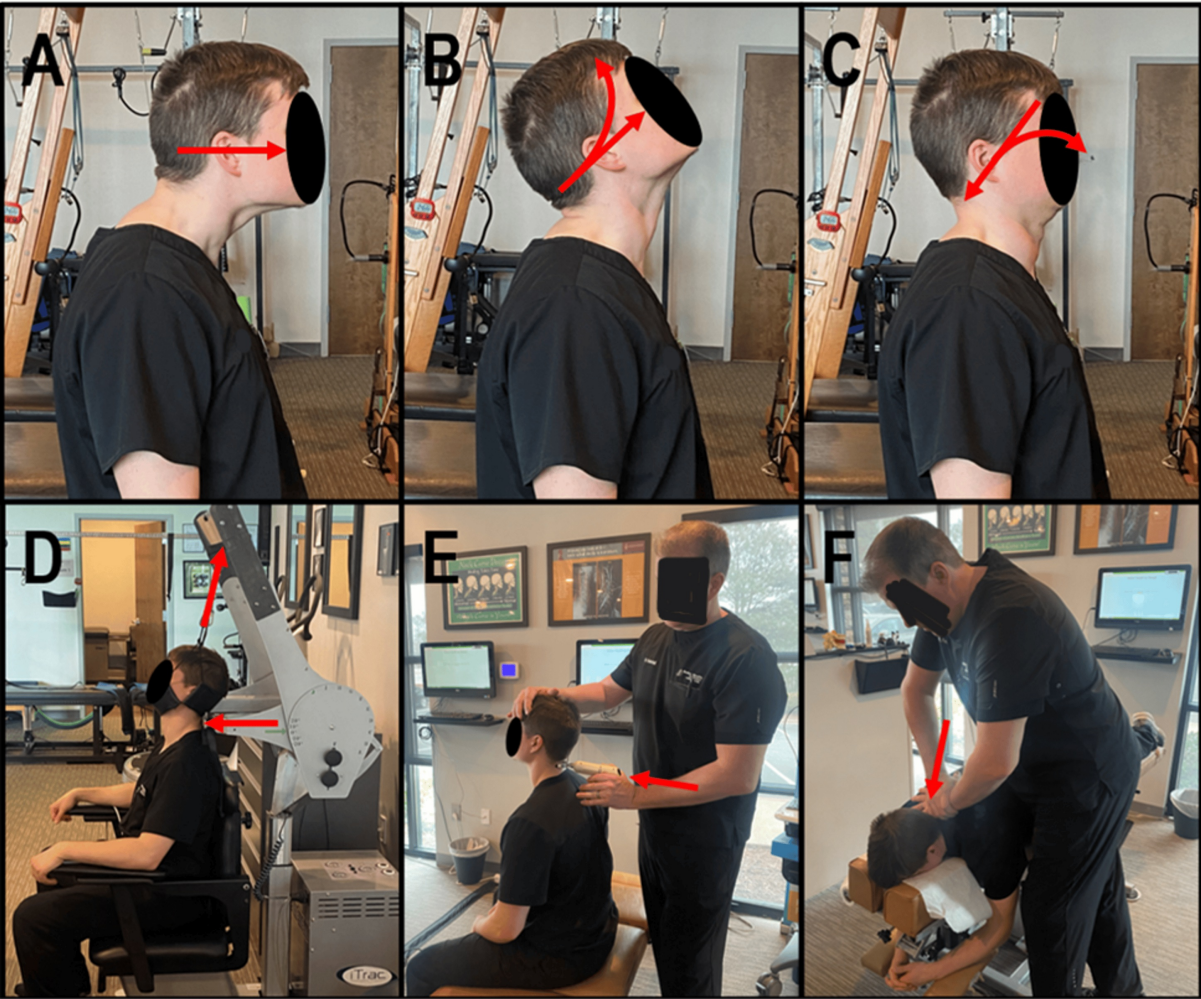
CBP® structural spinal rehabilitation therapeutic exercise, mechanical traction, and Mirror Image® spinal manipulative therapy. The red arrow represents prescribed movements/forces for structural spinal rehabilitation treatment using CBP® protocols. (A-C) Mirror Image® exercise: (A) maximum +TzH, (B) +TzH with maximum -RxH, and (C) -TzH and -TyH; (D) MI® cervical two-way traction combining +TyH and -TzH with +Tz mid cervical; and (E,F) Mirror Image® SMT: (E) seated MI +Tz mid cervical adjustment using the Impulse® manually assisted spinal manipulative thrust instrument and (F) Prone MI® +Tz mid-cervical SMT to induce lordosis CBP®: Chiropractic BioPhysics®; +TzH: maximum anterior head translation; -RxH: maximum head extension; -TyH: inferior head translation; MI®: Mirror Image®; +TyH: cervical distraction; SMT: spinal manipulative therapy

CBP® technique aims to normalize spinal alignment and biomechanics by applying Mirror Image® (MI®) spinal exercises, mechanical traction, and chiropractic adjustments to address musculature, connective tissue, and neurology, respectively. The patient completed 30 in-office sessions of CBP® MI spinal structural rehabilitation over nine weeks. The MI® spinal exercises involved the patient performing Fedorchuk lordosis-inducing procedure exercises, including the following steps: 1) maximum anterior head translation (+T_z_H), which creates lordosis of the upper cervical spine (Figure 2A); 2) while maintaining +T_z_H, maximum head extension (-RxH), which creates lordosis of the lower cervical spine (Figure 2B), and 3) while maintaining the -RxH, posterior and inferior head translation (-T_z_H and -TyH), which stabilizes the cervical curve and returns the head to the normal postural position (Figure 2C).

The final position is held for 10 seconds before relaxing for 10 repetitions and increasing to 50 repetitions (Figures 2A-2C). MI® exercises strengthen and lengthen muscles that have adapted to unhealthy posture to correct and maintain healthy spinal alignment and posture [11]. MI® traction was performed using iTrac Spine Remodeling System™ (Spine Remodeling Systems, LLC., Manhattan, KS), which is designed to safely apply spinal traction while maximizing patient comfort (Figure 2D). Two-way traction combines cervical distraction (+T_y_H) and posterior head translation (-T_z_H). It is applied using a chin-occiput harness and +T_z_ is applied to the mid-cervical vertebra. The treatment periods start at eight minutes and increase by two minutes with each visit until 16 minutes was reached. MI® traction allows for viscoelastic plastic deformation of spinal ligaments [11] and corrects the patient's abnormal posture by initiating muscle and ligament creep, creating permanent restorative change [12]. MI® spinal manipulative therapy (SMT) involved using the Impulse® adjusting instrument (Neuromechanical Innovations, Chandler, AZ), setting up the patient in the corrected position and applying a +T_z_ mid-cervical adjustment (Figure 2E). A mechanical drop table was also used for MI SMT (Figure 2F). MI® SMT improves ROM, reduces pain, and helps improve postural abnormalities [11].

The patient was advised to perform the above exercise daily, improve ergonomics and postural habits, and inform the treating physician if the symptoms return or worsen. The patient was compliant and consistent, missed no appointments, and performed all recommended postural exercises. No adverse effects or events were reported throughout or following care, and the patient reported high satisfaction with the results and outcome. The subject consented to the publication of this case, including all pictures and radiographs.

Results

Posttreatment Examination

Subjective outcomes: A posttreatment reexamination was performed in November 2020, following 30 visits over nine weeks. The patient was not treated within 24 hours of the reexamination to ensure there were no lingering effects of the prior treatment. The patient reported near resolution of the CNP and stiffness with disabling radiating pains into the middle back and extremities. The patient described his NP as a mild, occasional pain that he rated 2/10 on the neck and back pain NRS. Cervical compression orthopedic testing no longer caused neck or radiating spine pain.

Posttreatment SF-36 showed significant improvement in HRQoL measures. SF-36 demonstrated improvement in PF to 70/100, RP to 50/100, RE to 50/100, VT to 60/100, MH to 52/100, SF to 37.5/100, BP to 67.5/100, GH to 55/100, and ΔH at 75/100 (Table 1).

The patient reported significant improvement in cervical ROM. Visual ROM was also improved in all directions without pain or restriction. The patient was able to turn their head during normal activities of daily living, including driving and work, and was very happy with eliminating the CNP and stiffness. The patient stated he was no longer taking pain medications. The patient also reported a much better outlook with minimal depression symptoms reported. The patient's grip strength normalized bilaterally, measuring 45.3 kg on the left and 49.4 on the right.

Radiographic results: Posttreatment radiographs were performed following more than 24 hours between the last treatment and reevaluation. The radiographs were acquired by the same physician as the initial assessment, and all radiographic factors and techniques were duplicated. Following the initial 30 treatments, ARA C2-C7 improved by 15.4° from 14.7° to 30.1°, and T_z_ C2-C7 improved by 5 mm from 20.6 mm to 15.6 mm (Figure 1B). This is consistent with results from previous CBP® studies [10,13]. According to Harrison et al., this improvement in anterior translation brings the parameter within normal limits in the 2004 modeling study of the normal cervical spine parameters [14].

Six-Month Follow-Up Examination

The patient was unable to continue in-office treatments as he was attending college. After six months, when he returned from school in May 2021, an evaluation was performed in which no treatment had been performed in the interim. The patient's symptoms had not returned, and his outcome measures, including cervical ROM, grip strength, and HRQoL (Table 1), found no significant regression. ARA C2-C7 maintained improvement at 30.9°, and T_z_ C2-C7 improved 5 mm further to 10.6 mm (Figure 1C).

## Discussion

This report details the successful treatment of a 26-year-old male suffering from CNP. The patient had endured severe suffering with unsuccessful medical, surgical, and conservative therapies. Following improvements in cervical curvature (ARA C2-C7) and T_z_ C2-C7 using these structural spinal rehabilitation protocols, the patient experienced improved subjective and functional symptoms and PROs that were maintained after six months with no treatment.

NP is a major contributor to the global burden of disease (GBD) and is a significant source of disability and reduction in HRQoL measures. NP is a major cause of disability, and the pain will often force the patient to seek conservative and invasive procedures to continue to work [1-3]. Analgesics, OTC and prescribed medications, physical therapy, and more invasive procedures, including nerve ablation, minimally invasive surgery, and multilevel spinal fusion, are all described in medical literature to treat NP. Alterations of spinal alignment and posture cause abnormal tissue loads across multiple pain-sensitive structures [15]. This can lead to adult spine deformity (ASD) and the need for surgical intervention [16]. Further, spine surgery is associated with adjacent segment degeneration and disease [17], leading to worsening GBD for NP and neck-related disability [1,8]. Few conservative options are reported in the literature with successful short-term and long-term improvements in HRQoL [18,19], as reported in this case.

While published studies show that most cervical spine fusions are considered safe, failures and complications are reported nonetheless [6,7,18,19]. Cervical spine fusion complications and failures are measured via subjective and objective outcome measure reporting. These include PROs and other outcome measures, such as the pain NRS and the SF-36. Objective measures to determine the cause of the complication or failure include advanced imaging, plain film radiography, and nerve conduction velocity studies. Biopsy, discogram, and ultrasound can be used to determine the level of disease, infection, and vascular abnormalities. Interventions must be undertaken with a cost-benefit analysis of the measure, and the astute clinician will use minimally invasive and cost-effective outcomes before moving to more invasive and much more costly assessments [2-3,6-8]. Maintenance of cervical lordosis is an important part of predicting postoperative failures [5,14,20].

Successful conservative treatment reports following failed cervical spine surgeries are rare in scientific literature. Medications such as opioids, nonsteroidal anti-inflammatory drugs, muscle relaxants, antidepressants, corticosteroids, and others are much more common than physical, manual, and other conservative therapies, but there are few long-term studies. Studies show that one-year follow-ups with traditional physical therapies (e.g., supervised isometric exercises and balance and proprioceptive training) were superior to ultrasound and heat therapy, which only appeared to have short-term analgesic properties [6-8,17-21]. It is rare to find a one-year or longer follow-up with any current nonsurgical interventions for failed neck surgery in the literature [22]. Further, long-term, randomized, or nonrandomized case-controlled trials comparing upper cervical fusion versus conservative therapies versus no treatment are not found in the literature.

This conservative structural spinal rehabilitation consists of a therapy regimen including measurable, specific MI spinal exercises, mechanical traction, and SMT focused on improving spinal alignment and postural balance [11,13,23,24] to help reduce abnormal spinal, paraspinal and extraspinal tissue loads to improve pain and function. In scientific literature, CBP® studies demonstrate short- and long-term improvements in subjective PROs and HRQoL measures following correction of spinal alignment and posture [10,11,13,23]. Structural rehab protocol treatments have shown improvements in sagittal and coronal spinal alignments and postural alignments using repeatable, reliable, and valid assessments showing improved PROs across many various spinal and extraspinal conditions [10,25-27]. Spinal radiographic analysis allows physicians, surgeons, and therapists to safely [28-31] make differential diagnoses using spinal alignment measurements compared to established average and ideal values and prescribe specific, effective therapies [12,32-34].

This case study is limited in its ability to draw conclusions about correlation and causation, or apply the findings to a broader spectrum of varying demographics. As is true for case studies, this study is further limited by sampling bias. Clinical trials involving patients with cervical spinal fusions that fail to resolve neck problems and spinal alignment, structural spinal rehabilitation, medical and surgical management, and control groups with long-term follow-ups are needed.

## Conclusions

This case report showed improvements in subjective and objective measures following conservative structural spinal rehabilitation for a patient with failure to improve following medication and upper cervical fusion surgery. This case adds to the literature the potential treatment of PSPS and failed surgical syndromes using a conservative multimodal approach. Conservative structural spinal rehabilitation may be an effective option to help improve PSPS pain, dysfunction, and disability in a patient with reduced cervical lordosis, poor spinal mechanics, and prior failed surgical fusion. Sagittal balance of the spine and posture is a desirable clinical outcome. Improving ASD reduces healthcare related costs and lessens GBD. These structural protocols offer an inexpensive, reliable, repeatable and valid treatment protocol with a significant amount of literature across multiple conditions with positive subjective and objective outcomes. Further research on this unique population is needed to validate the feasibility of the treatment approach described herein.

## References

[1] Safiri S, Kolahi AA, Hoy D (2020). Global, regional, and national burden of neck pain in the general population, 1990-2017: systematic analysis of the Global Burden of Disease Study 2017. BMJ.

[2] Lubelski D, Alvin MD, Nesterenko S, Sundar SJ, Thompson NR, Benzel EC, Mroz TE (2016). Correlation of quality of life and functional outcome measures for cervical spondylotic myelopathy. J Neurosurg Spine.

[3] Zuckerman SL, Devin CJ (2020). Outcomes and value in elective cervical spine surgery: an introductory and practical narrative review. J Spine Surg.

[4] Passias PG, Pierce KE, Passano B (2021). What are the major drivers of outcomes in cervical deformity surgery?. J Craniovertebr Junction Spine.

[5] Zhang JT, Li JQ, Niu RJ, Liu Z, Tong T, Shen Y (2017). Predictors of cervical lordosis loss after laminoplasty in patients with cervical spondylotic myelopathy. Eur Spine J.

[6] Verla T, Xu DS, Davis MJ (2021). Failure in cervical spinal fusion and current management modalities. Semin Plast Surg.

[7] Andresen AK, Paulsen RT, Busch F, Isenberg-Jørgensen A, Carreon LY, Andersen MØ (2018). Patient-reported outcomes and patient-reported satisfaction after surgical treatment for cervical radiculopathy. Global Spine J.

[8] Cauthen JC, Kinard RE, Vogler JB (1998). Outcome analysis of noninstrumented anterior cervical discectomy and interbody fusion in 348 patients. Spine (Phila Pa 1976).

[9] Amaral CA, Amaral TL, Monteiro GT, Vasconcellos MT, Portela MC (2019). Hand grip strength: reference values for adults and elderly people of Rio Branco, Acre, Brazil. PLoS One.

[10] Oakley PA, Kallan S, Harrison DE (2022). Improving the pediatric cervical lordosis: a review of Chiropractic Biophysics® case reporting. J Contemp Chiropr.

[11] Oakley PA, Harrison DD, Harrison DE (2005). Evidence-based protocol for structural rehabilitation of the spine and posture: review of clinical biomechanics of posture (CBP®) publications. J Can Chiropr Assoc.

[12] Harrison DE, Holland B, Harrison DD, Janik TJ (2002). Further reliability analysis of the Harrison radiographic line-drawing methods: crossed ICCs for lateral posterior tangents and modified Risser-Ferguson method on AP views. J Manipulative Physiol Ther.

[13] Oakley PA, Ehsani NN, Moustafa IM, Harrison DE (2021). Restoring cervical lordosis by cervical extension traction methods in the treatment of cervical spine disorders: a systematic review of controlled trials. J Phys Ther Sci.

[14] Harrison DD, Harrison DE, Janik TJ, Cailliet R, Ferrantelli JR, Haas JW, Holland B (2004). Modeling of the sagittal cervical spine as a method to discriminate hypolordosis: results of elliptical and circular modeling in 72 asymptomatic subjects, 52 acute neck pain subjects, and 70 chronic neck pain subjects. Spine (Phila Pa 1976).

[15] Figas G, Kostka J, Pikala M, Kujawa JE, Adamczewski T (2024). Analysis of clinical pattern of musculoskeletal disorders in the cervical and cervico-thoracic regions of the spine. J Clin Med.

[16] Naresh-Babu J, Kwan KY, Wu Y (2023). AO spine adult spinal deformity patient profile: a paradigm shift in comprehensive patient evaluation in order to optimize treatment and improve patient care. Global Spine J.

[17] Cho SK, Riew KD (2013). Adjacent segment disease following cervical spine surgery. J Am Acad Orthop Surg.

[18] Papalia GF, Russo F, Vadalà G (2023). Non-invasive treatments for failed back surgery syndrome: a systematic review. Global Spine J.

[19] Hirpara KM, Butler JS, Dolan RT, O'Byrne JM, Poynton AR (2012). Nonoperative modalities to treat symptomatic cervical spondylosis. Adv Orthop.

[20] Hu X, Ohnmeiss DD, Zigler JE, Guyer RD, Lieberman IH (2015). Restoration of cervical alignment is associated with improved clinical outcome after one and two level anterior cervical discectomy and fusion. Int J Spine Surg.

[21] Cho JH, Lee JH, Song KS (2017). Treatment outcomes for patients with failed back surgery. Pain Physician.

[22] Oakley PA, Harrison DE (2018). Alleviation of pain and disability in a post-surgical C4-C7 total fusion patient after reducing a lateral head translation (side shift) posture: a CBP(®) case report with a 14 year follow-up. J Phys Ther Sci.

[23] Harrison DE, Oakley PA (2022). An introduction to Chiropractic BioPhysics® (CBP®) technique: a full spine rehabilitation approach to reducing spine deformities. Complementary Therapies.

[24] Oakley PA, Ehsani NN, Moustafa IM, Harrison DE (2020). Restoring lumbar lordosis: a systematic review of controlled trials utilizing Chiropractic Bio Physics(®) (CBP(®)) non-surgical approach to increasing lumbar lordosis in the treatment of low back disorders. J Phys Ther Sci.

[25] Oakley PA, Kallan SZ, Harrison DE (2022). Structural rehabilitation of the cervical lordosis and forward head posture: a selective review of Chiropractic BioPhysics(®) case reports. J Phys Ther Sci.

[26] Oakley PA, Kallan S, Harrison DE (2022). Structural rehabilitation of the lumbar lordosis: a selective review of CBP® case reports. J Contemp Chiropr.

[27] Oakley PA, Harrison DE (2018). Reducing thoracic hyperkyphosis subluxation deformity: a systematic review of Chiropractic BioPhysics® methods employed in its structural improvement. J Contemp Chiropr.

[28] Marcus CS (2016). Destroying the linear no-threshold basis for radiation regulation: a commentary. Dose Response.

[29] Cuttler JM (2020). Application of low doses of ionizing radiation in medical therapies. Dose Response.

[30] Oakley PA, Harrison DE (2018). Radiophobia: 7 reasons why radiography used in spine and posture rehabilitation should not be feared or avoided. Dose Response.

[31] Oakley PA, Cuttler JM, Harrison DE (2018). X-Ray imaging is essential for contemporary chiropractic and manual therapy spinal rehabilitation: radiography increases benefits and reduces risks. Dose Response.

[32] Janik TJ, Harrison DD, Cailliet R, Troyanovich SJ, Harrison DE (1998). Can the sagittal lumbar curvature be closely approximated by an ellipse?. J Orthop Res.

[33] Harrison DE, Cailliet R, Harrison DD, Janik TJ, Holland B (2001). Reliability of centroid, Cobb, and Harrison posterior tangent methods: which to choose for analysis of thoracic kyphosis. Spine (Phila Pa 1976).

[34] Harrison DE, Harrison DD, Cailliet R, Janik TJ, Holland B (2001). Radiographic analysis of lumbar lordosis: centroid, Cobb, TRALL, and Harrison posterior tangent methods. Spine (Phila Pa 1976).

